# Clinical utility of the “balloon lever technique” in the right inferior pulmonary vein cryoballoon ablation

**DOI:** 10.1002/joa3.12801

**Published:** 2022-12-19

**Authors:** Yasushi Wakabayashi, Masanori Kobayashi, Tomohide Ichikawa, Takashi Koyama, Hidetoshi Abe

**Affiliations:** ^1^ Department of Cardiovascular Medicine Matsumoto Kyoritsu Hospital Matsumoto Nagano Japan

**Keywords:** atrial fibrillation, balloon lever technique, cryoballoon ablation, pulmonary vein isolation, right inferior pulmonary vein

## Abstract

**Background:**

The acute success rate of pulmonary vein isolation (PVI) with cryoballoon (CB) is reported to be lower in the right inferior pulmonary vein (RIPV). This study aimed to investigate the utility of the “balloon lever technique (BLT)” for RIPV CB ablation.

**Methods:**

We retrospectively studied consecutive patients who underwent CB‐PVI for atrial fibrillation between February 21, 2020 and June 3, 2022. RIPV cryoablation was performed according to a specific protocol. The patients underwent RIPV cryoablation using the conventional method. If the method was found ineffective, BLT cryoablation was performed. The acute success rate of RIPV CB ablation was examined. We also investigated the RIPV isolation rate and procedural parameters during conventional and BLT cryoablation.

**Results:**

Ninety‐three patients were included in the analysis. RIPV isolation was achieved in 89.2% (83/93) of the patients using conventional method and subsequent BLT cryoablation. Meanwhile, 68 patients underwent BLT cryoablation because the conventional method was ineffective. RIPV was isolated with BLT in 85.3% (58/68) of patients. Additionally, BLT was found to be superior to conventional cryoablation in terms of nadir balloon temperature, freezing time, and thawing time to a specific temperature in patients who underwent both conventional and BLT cryoablations.

**Conclusions:**

BLT is useful in RIPV cryoablation when the conventional method is ineffective. BLT cryoablation may be helpful, mainly because of the BLT‐mediated contact of the balloon with the bottom of the RIPV, which leads to optimal RIPV occlusion.

## Graphical abstract

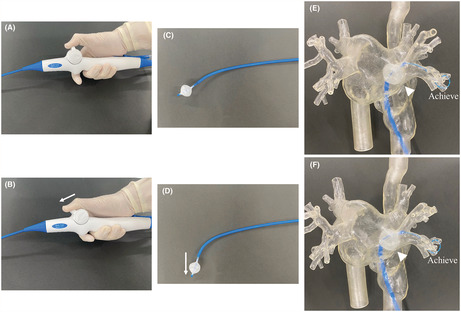

## 1 INTRODUCTION

Pulmonary vein (PV) isolation (PVI) using cryoballoon (CB) technology has become an established therapeutic strategy for the treatment of atrial fibrillation (AF).^1^ PV reconnection is usually the main cause of AF recurrence. CBs are effective and associated with lower rates of recurrent AF, owing to the higher durability of electrical PVI.^2^ However, the incidence of right inferior PV (RIPV) reconnections was reported to be higher than that of the other three PVs.^3^ The acute CB‐PVI success rate was also lower in RIPVs because of the anatomical difficulty in achieving complete RIPV occlusion with CB.^4^ Previous studies have reported several techniques for RIPV occlusion or CB ablation,^5^, ^6^ although the “balloon lever technique (BLT)”, which is a relatively simple procedure, has not been previously reported.

This study aimed to investigate the clinical utility of BLT in RIPV CB ablation.

## 2 METHODS

### 2.1 Study population

We retrospectively studied consecutive patients who underwent CB‐PVI between February 21, 2020 and June 3, 2022, at Matsumoto Kyoritsu Hospital. If RIPV cryoablation was performed against the protocol described below, patients were excluded from the analysis. The study protocol was approved by the ethics committee of Matsumoto Kyoritsu Hospital.

### 2.2 CB ablation procedure

Transesophageal echocardiography was performed before the procedure to rule out the potential for a left atrial thrombus. Preprocedural cardiac computed tomography (CT) was also performed to create three‐dimensional anatomical models of the left atrium (LA) and analyze the PV anatomy. Surface electrocardiograms and intracardiac electrograms were continuously monitored and stored using a Bard LabSystem Pro Electrophysiology recording system (Boston Scientific). All patients were completely sedated and fitted with artificial respirators using laryngeal masks. A 5000 U of heparin bolus was administered intravenously after venous access was obtained. Heparin was infused intermittently to maintain an activated clotting time >300 s. A 20‐polar superior vena cava–right atrium–coronary sinus electrode catheter (BeeAT; Japan Lifeline) was inserted through the right jugular vein into the coronary sinus. A single transseptal puncture was performed under fluoroscopic guidance. For the puncture, periprocedural intracardiac echocardiography, a radiofrequency needle (RF Needle; Japan Lifeline), and an 8.5‐Fr long sheath (SL‐0; Abbott Laboratories) were used, followed by the insertion of a 15‐Fr steerable sheath (FlexCath Advance; Medtronic), which was continuously flushed with heparinized saline. A 28‐mm fourth‐generation CB (Arctic Front Advance Pro; Medtronic) was advanced to the LA through a circular mapping catheter (Achieve Mapping Catheter; Medtronic) used as a guidewire. The Achieve catheter was advanced and positioned inside each PV to support the CB and record PV potentials. The CB was then inflated and advanced into the PV ostium. If the selective contrast injection to the PVs demonstrated contrast retention with negligible leakage and the shape of the PV was discernible, PV occlusion was considered optimal. Thus, the optimal occlusion comprised occlusion grades 3 and 4. Occlusion grade was a semiquantitative grading: grade 4 = excellent (full retention of contrast medium without visible outflow) to grade 1 = very poor (immediate rapid outflow from the PV).^7^ We also confirmed optimal PV occlusion without contrast media using a pressure waveform and a change in pressure at the tip of the balloon catheter. If the pressure curve converted from left atrial to pulmonary artery pressure with an increased average pressure value, PV occlusion was considered optimal.^8^, ^9^ Once optimal occlusion was documented, cryoablation was initiated, for which the standard freeze duration was 180 s. If a balloon temperature ≤−40°C could not be obtained within 60 s, the freezing duration was extended to 240 s. Cryoablation was initiated from the left superior PV, moving on to the left inferior PV, RIPV, and the right superior PV. Phrenic nerve function was monitored using continuous phrenic nerve pacing during cryoablation of the right PV to avoid phrenic nerve injuries. If a significant decrease in the amplitude of the diaphragmatic compound motor action potentials (CMAPs) was observed, freezing was terminated with active deflation. Additionally, if the balloon temperature reached −60°C or the esophageal temperature reached 10°C, freezing was terminated with passive deflation. Radiofrequency touch‐up ablation was performed using an irrigated‐tip catheter (THERMOCOOL SMARTTOUCH SF Catheter; Johnson & Johnson or TactiCath Contact Force Ablation Catheter, Sensor Enabled; Abbott Laboratories) if PVI was not achieved despite several times of CB ablation. Acute procedural success was defined as electrical isolation of all PVs during isoproterenol and adenosine triphosphate infusion. Procedure time was defined as the time from the puncture to the final confirmation of PVI.

### 2.3 Ablation of the RIPV and BLT

**FIGURE 1:**
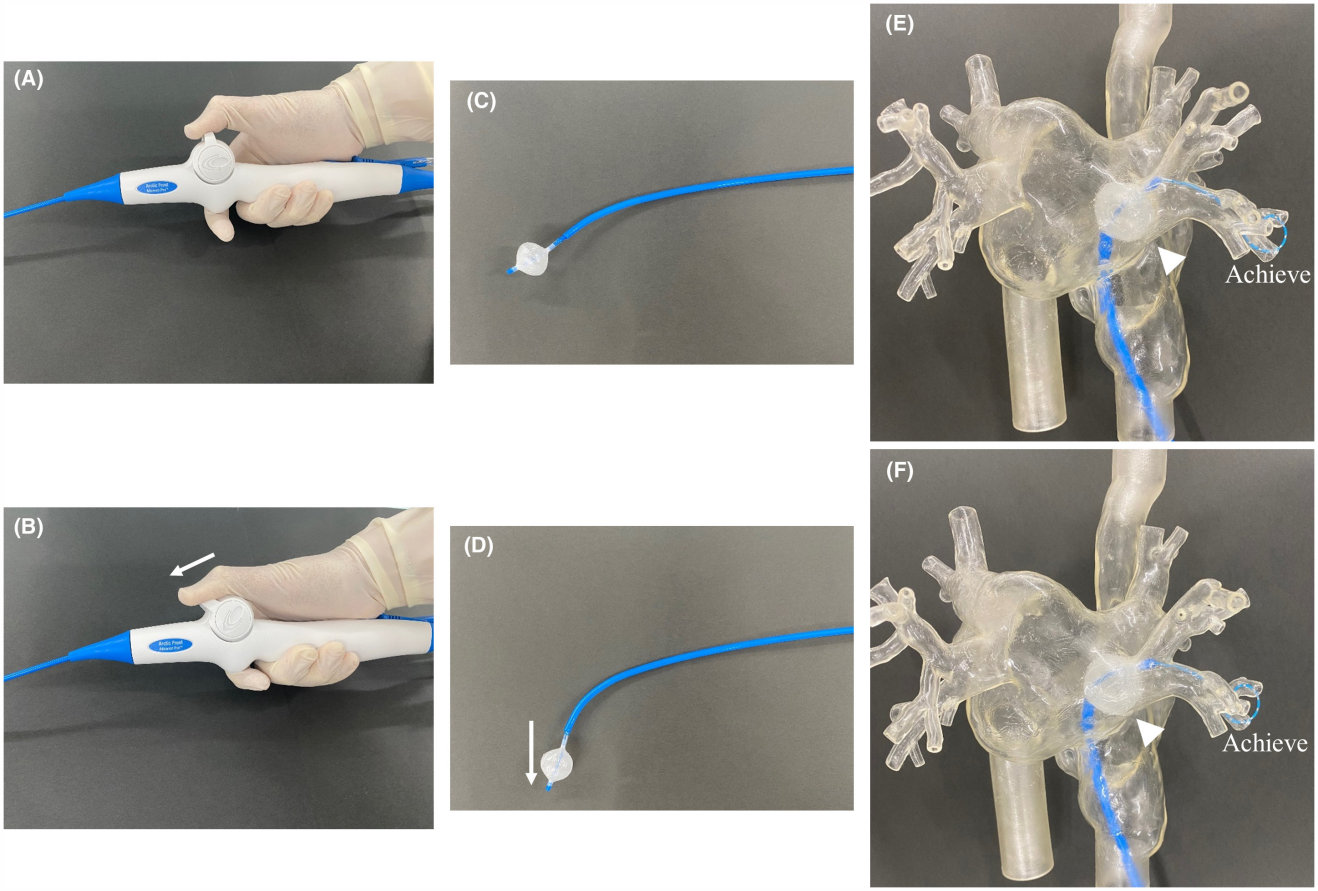
Balloon lever technique (BLT). (A) The initial position of the balloon lever. (B) The balloon lever is pressed forward, leading to the downward shift of the balloon, as shown in (D). (C) The initial position of the cryoballoon (CB). (D) The downward shift of the CB that is caused by the forward displacement of the lever, as shown in (B). (E) The model of the left atrium. The achieve catheter is inserted into the inferior branch of the right inferior pulmonary vein (RIPV). The CB is moved forward with the conventional method. A space is observed between the CB and the bottom of the RIPV (white arrowhead). (F) The model of the left atrium. The CB is moved towards the RIPV using the BLT. The CB is pressed to the bottom of the RIPV antrum, leading to the disappearance of the space (white arrowhead).

In our hospital, RIPV CB ablation is performed according to a specific protocol. Achieve catheter was inserted into the inferior branch of the RIPV. The CB was inflated, advanced into the ostium, and positioned coaxially with RIPV. The FlexCath Advance was bent appropriately for optimal RIPV occlusion. If optimal occlusion was not achieved with leakage from the inferior site of the RIPV, the sheath was pulled downwards for occlusion. Cryoablation was performed when optimal RIPV occlusion was confirmed, which is the conventional method. However, the freezing process was terminated prematurely if rapid cooling (indicated by the balloon temperature reaching ≤−40°C within 60 s) was not achieved. If optimal RIPV occlusion was not obtained, rapid cooling was not achieved, or the RIPV was not isolated with conventional cryoablation, the balloon lever was moved forward (Figure 1A,B). The balloon was maneuvered downward (Figure 1C,D) and pressed to the bottom of the RIPV antrum (Figure 1E,F), which is a typical process of the “BLT”. A BLT was used to attain optimal occlusion, and cryoablation was performed. Several BLT CB ablations were allowed if RIPV isolation was not achieved. The preceding process indicated a specific protocol developed in our hospital. If RIPV was not isolated using this protocol, additional ablation, including touch‐up ablation, was performed at the operator's discretion.

We examined the acute success rate of RIPV CB ablation performed according to the hospital protocol. Furthermore, we investigated the RIPV isolation rate and procedural parameters during conventional and BLT cryoablation.

**FIGURE 2:**
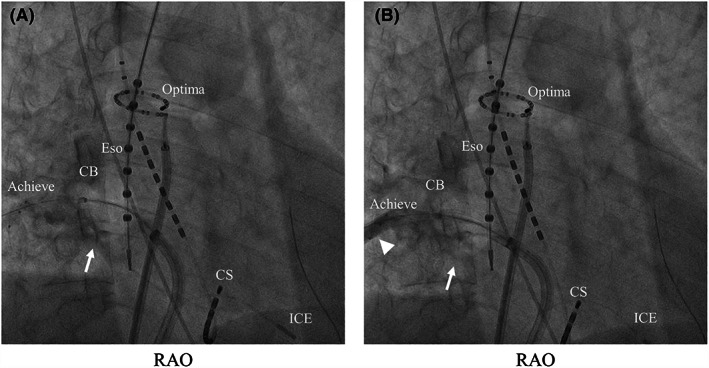
Right inferior pulmonary vein (RIPV) cryoballoon (CB) ablation with conventional method and balloon lever technique (BLT) in a representative patient. The achieve catheter is inserted into the inferior branch. (A) Massive leakage of the contrast medium is observed at the inferior site of the RIPV (white arrow). The shape of the RIPV could not be confirmed due to the flushing of the contrast medium. (B) Optimal RIPV occlusion is achieved with the BLT. The CB is shifted downward and pressed to the bottom of the RIPV (white arrow). A selective contrast injection to the RIPV shows contrast retention, and the shape of the RIPV could be discerned (white arrowhead). The optima is positioned in the superior vena cava. An intracardiac echocardiography catheter (ICE) is positioned in the right ventricle. CS, coronary sinus; Eso, esophageal temperature monitoring catheter; RAO, right anterior oblique projection.

Figure 2 shows the RIPV cryoablation using BLT in a representative patient.

### 2.4 Clinical data and measurements

The patient data was obtained from medical records and was retrospectively analyzed. Paroxysmal AF was defined as recurrent AF that terminated spontaneously and lasted ≤7 days, whereas persistent AF was defined as AF that lasted >7 days. Heart failure was defined according to the criteria of the Framingham Heart Study.^10^ Hypertensive patients were defined as those with a systemic arterial pressure >140/90 mmHg or those already taking antihypertensive drugs. Patients with diabetes were defined as those with a fasting plasma glucose level >126 mg/dl, a random plasma glucose level ≥200 mg/dl, a hemoglobin A1c level ≥6.5%, or those undergoing therapeutic interventions, such as oral drugs or insulin therapy. A stroke was defined as an episode of acute neurological dysfunction caused by ischemia or hemorrhage. The left atrial dimension and left ventricular ejection fraction were measured using the standard echocardiographic techniques. The anatomical parameters of the LA and RIPV were investigated using preprocedural contrast‐enhanced CT images as described previously.^11^

### 2.5 Statistical analysis

Continuous variables are expressed as mean ± standard deviation for normally distributed variables or median (interquartile range) for non‐parametric variables. The normality of the data was assessed using the Shapiro–Wilk test. Comparisons for normally distributed variables were performed with paired or unpaired *t*‐test. Non‐parametric variables were analyzed with the Mann–Whitney *U*‐test. Categorical data are presented as numbers (%) and were analyzed using the chi‐square test. Fisher's exact test was used to compare categorical variables with expected values <5. A value of *p* < .05 was considered statistically significant. Data were analyzed using SPSS version 19 (SPSS, Inc.).

## 3 RESULTS

### 3.1 Baseline characteristics

We identified 100 consecutive patients who underwent CB‐PVI for AF. Seven patients were excluded because RIPV CB ablation was not performed according to the protocol. Achieve catheter could not be inserted into the inferior branch in these two patients. Despite ineffective conventional ablations, BLT CB ablation was not performed in five patients. Finally, 93 patients were included in this analysis.

The baseline characteristics and procedural parameters of the patients are summarized in Table 1. The median patient age was 74 years, and 64.5% of the patients were male. Paroxysmal AF was observed in 81.7% (76/93) of patients, whereas persistent AF was observed in 18.3% (17/93) of patients. The percentage of patients with CHADS_2_ score ≥2 was 48.4% (45/93), and that of those with CHA_2_DS_2_VASc score ≥2 was 77.4% (72/93). A single RIPV CB ablation was applied in 64.5% (60/93) of patients, two CB ablations were applied in 26.9% (25/93), and three or more CB ablations were applied in 8.6% (8/93). Thus, 91.4% (85/93) of the patients underwent two or fewer CB applications. Touch‐up RIPV ablation was performed in 8.6% (8/93) of the patients.

**TABLE 1:** Baseline characteristics and procedural parameters.

Variables	All
	*n* = 93
Age (years)	74.0 (67.5–79.0)
Men	60 (64.5)
Paroxysmal atrial fibrillation	76 (81.7)
Persistent atrial fibrillation	17 (18.3)
Coronary artery disease	19 (20.4)
Congestive heart failure	21 (22.6)
Hypertension	47 (50.5)
Diabetes mellitus	26 (28.0)
Stroke	7 (7.5)
CHADS_2_ score ≥2	45 (48.4)
CHA_2_DS_2_VASc score ≥2	72 (77.4)
Left atrial dimension (mm)	39.7 ± 5.74
Left ventricular ejection fraction (%)	65.9 ± 10.0
Single RIPV cryoballoon application	60 (64.5)
Two RIPV cryoballoon applications	25 (26.9)
Three or more RIPV cryoballoon applications	8 (8.6)
Decrease of the CMAP amplitude during RIPV cryoballoon ablation	2 (2.2)
Touch‐up ablation for RIPV	8 (8.6)
Procedure time (min)	94.0 (85.5–108.5)
Fluoroscopy time (min)	34.0 (28.0–43.5)

*Note*: Values are mean ± SD, number of patients (%) or median (interquartile range).

Abbreviations: CMAP, compound motor action potential; RIPV, right inferior pulmonary vein; SD, standard deviation.

### 3.2 Flowchart of RIPV CB ablation

**FIGURE 3:**
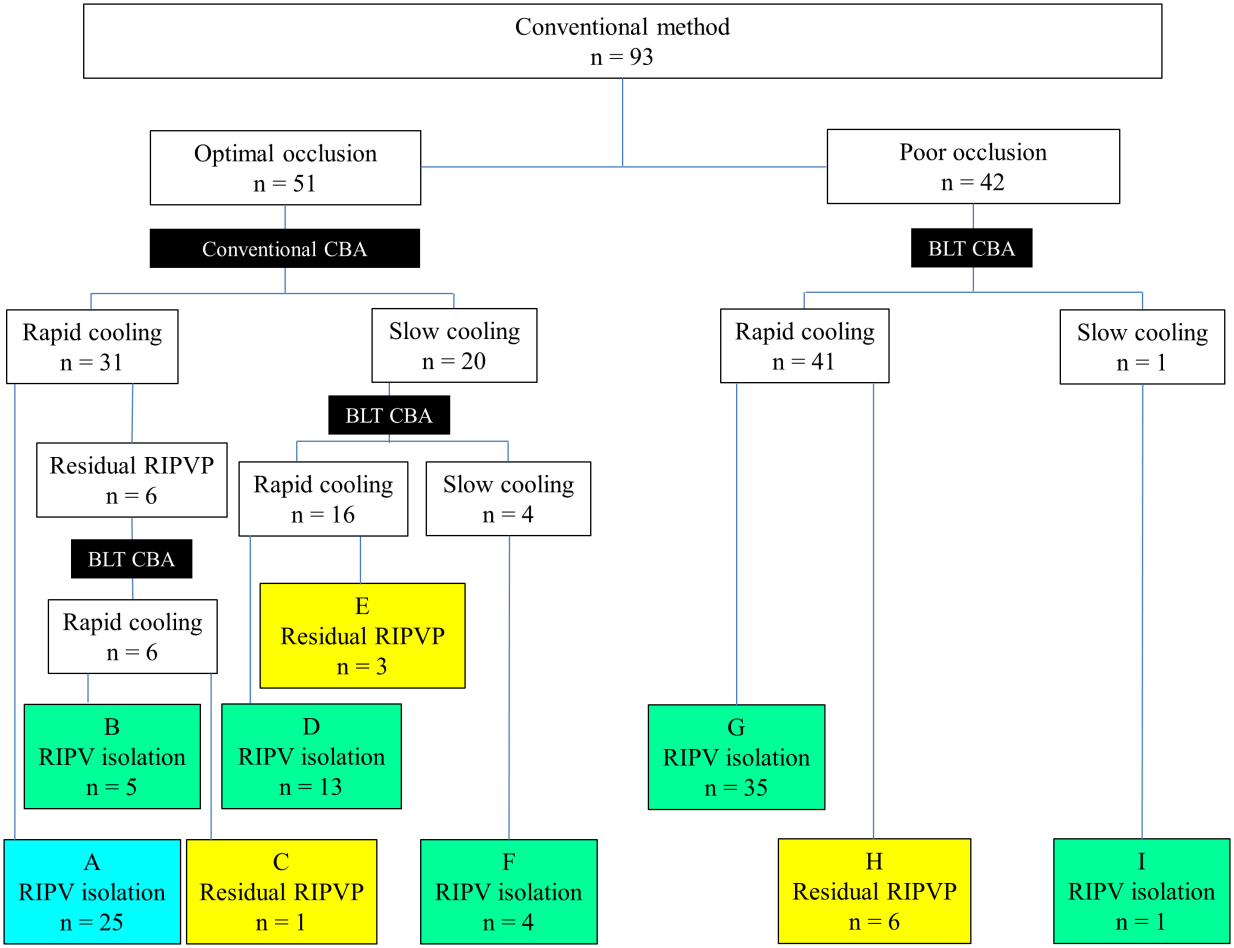
Flowchart summarizing the right inferior pulmonary vein (RIPV) cryoballoon ablation (CBA) and the distribution of the patients. The blue panel indicates the patients showing successful RIPV isolation with the initial conventional CBA. In contrast, the green panels indicate the patients showing RIPV isolation with the balloon lever technique (BLT) CBA. The yellow panels indicate the patients in whom RIPV was not isolated using the hospital protocol.

**FIGURE 4:**
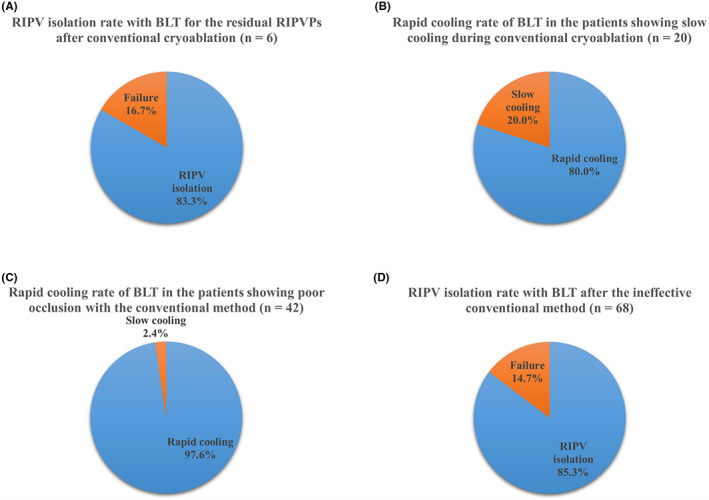
Effectiveness of balloon lever technique (BLT) after the ineffective conventional method. (A) Right inferior pulmonary vein (RIPV) isolation rate with BLT for the residual RIPV potentials (RIPVPs) after conventional cryoablation. (B) Rapid cooling rate of BLT in the patients showing slow cooling during conventional cryoablation. (C) Rapid cooling rate of BLT in the patients showing poor occlusion with the conventional method. (D) RIPV isolation rate with BLT after the ineffective conventional method.

**FIGURE 5:**
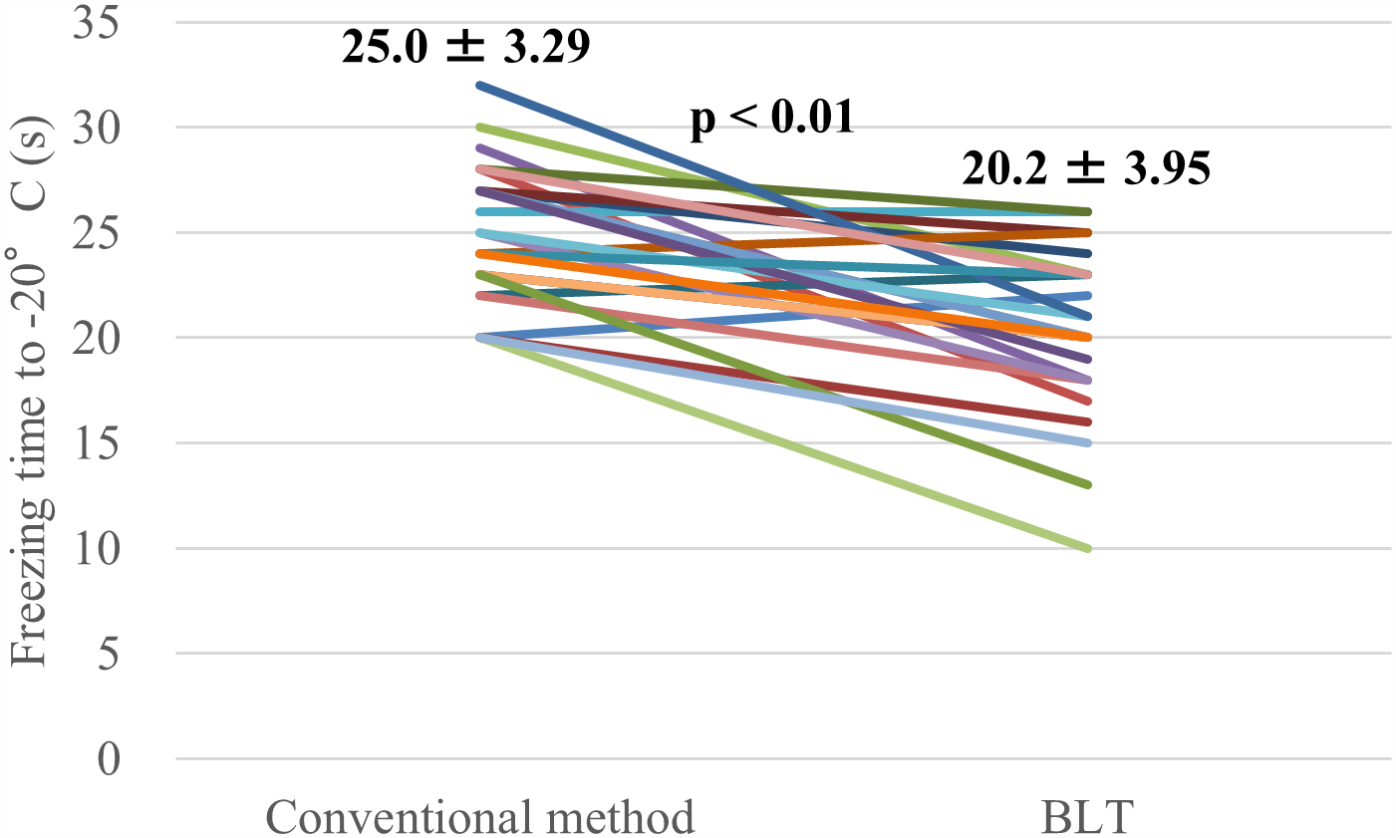
Comparison of the freezing time to −20°C between the initial conventional and the subsequent balloon lever technique (BLT) cryoablations (*n* = 26).

**FIGURE 6:**
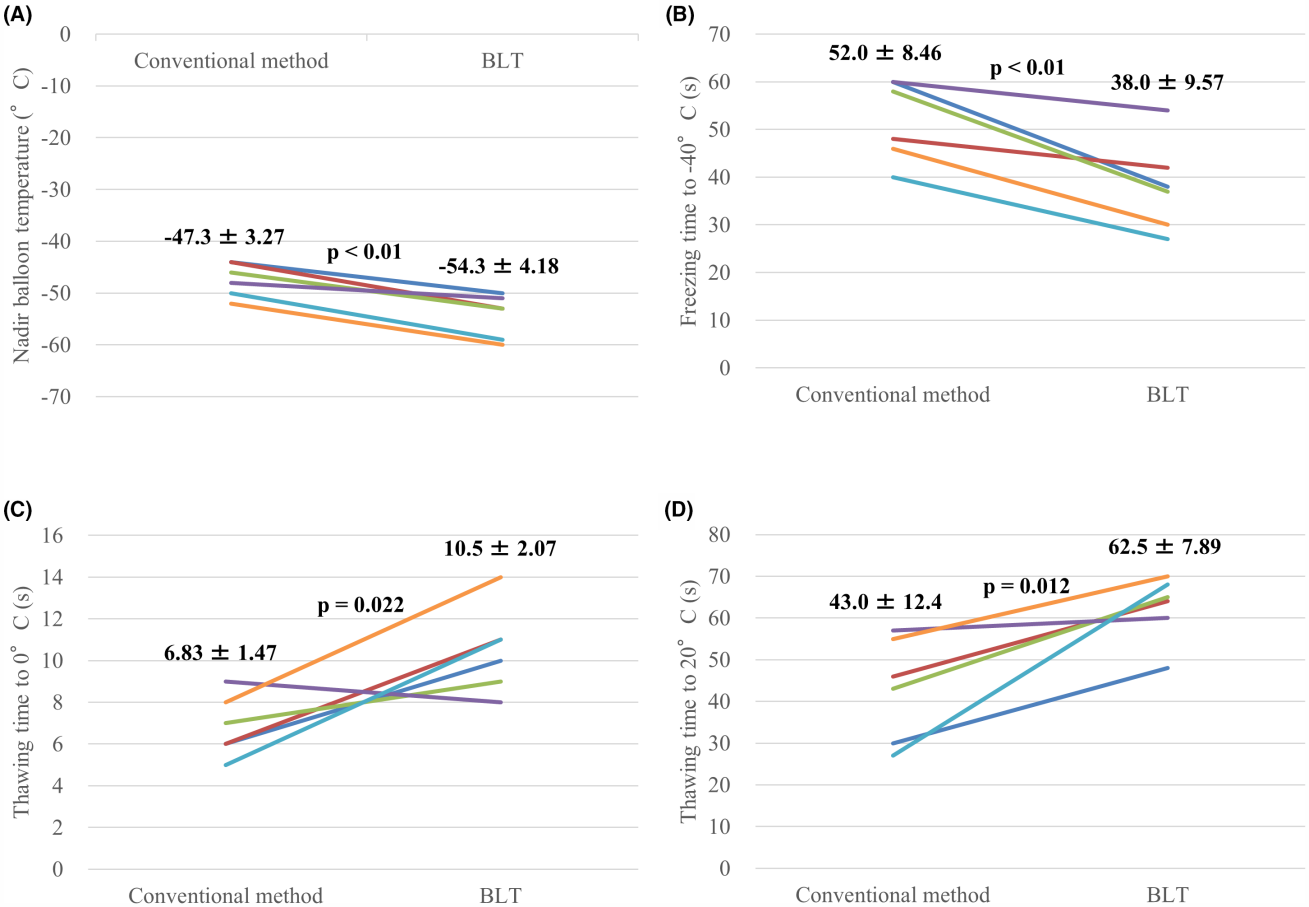
Comparisons of the nadir balloon temperature (A), freezing time to −40°C (B), thawing time to 0°C (C) and 20°C (D) between the initial conventional and the subsequent balloon lever technique (BLT) cryoablations (*n* = 6).

A flowchart of RIPV CB ablation and patient distribution are summarized in Figure 3. Optimal occlusion was targeted for all patients using conventional methods and was achieved in 51 patients but not in 42 patients. Conventional cryoablation was performed in 51 patients with optimal occlusion. Rapid cooling was observed in 31 of these patients, with RIPV isolation successful in 25 (panel A). In the remaining six patients, RIPV was not isolated using conventional cryoablation, and BLT was used. Rapid cooling was observed in all six patients. RIPV was isolated in five patients (panel B) but not in one patient (panel C) (Figure 4A). Among the 51 patients showing optimal occlusion using the conventional method, slow cooling was observed in 20 of them, for whom BLT cryoablation was performed. Rapid cooling was observed in 16 patients (Figure 4B), and RIPV was isolated in 13 of these patients (panel D), whereas it was not isolated in three patients (panel E). The other four patients showed slow cooling with BLT cryoablation, although RIPV was isolated (panel F). Meanwhile, BLT cryoablation was performed in 42 patients who demonstrated poor occlusion using the conventional method. Among these patients, rapid cooling was observed in 41 patients (Figure 4C), and subsequent RIPV isolation was accomplished in 35 patients (panel G) but not in 6 patients (panel H). One patient showed slow cooling with BLT cryoablation, even though the RIPV was isolated (panel I). Finally, 68 patients underwent BLT cryoablation because the conventional method was considered ineffective owing to poor occlusion, slow cooling, or residual RIPV potentials (green and yellow panels). RIPV was isolated with the BLT in 85.3% (58/68) of the patients (green panels) (Figure 4D), although five patients showed slow cooling. Furthermore, both conventional and subsequent BLT CB ablations were performed in 26 patients who were distributed across panels B–F. However, the initial conventional freezing was stopped after 60 s owing to slow cooling in the 20 patients belonging to panels D–F. A comparison of freezing time to −20°C between the initial conventional method and subsequent BLT in the 26 patients is shown in Figure 5. Additionally, comparisons of nadir balloon temperature, freezing time to −40°C, thawing time to 0 and 20°C between the initial conventional method and subsequent BLT in the six patients are shown in Figure 6, in which the initial conventional freezing was not stopped prematurely. The freezing time to −20°C during BLT was found to be significantly shorter than that during conventional CB ablation (Figure 5). The nadir balloon temperature was significantly lower and freezing time to −40°C was significantly shorter during BLT (Figure 6A,B). Thawing time to 0 and 20°C during BLT was significantly longer than during conventional cryoablation (Figure 6C,D).

Contrast injection was performed to estimate PV occlusion in 37.6% (35/93) of patients. The remaining patients underwent cryoablation without contrast media. Therefore, the occlusion grade was examined in 35 patients. Occlusion grade 4 was observed in 17.1% (6/35) of patients using the conventional method, of which both rapid cooling and RIPV isolation were observed in five patients (panel A). Occlusion grade 3 was observed in 25.7% (9/35) of patients using the conventional method, of which both rapid cooling and RIPV isolation were observed in five patients (panel A). The remaining five patients with occlusion grades 3 or 4 underwent subsequent BLT cryoablation. Meanwhile, occlusion grades 1 or 2 were observed in 57.1% (20/35) of patients using the conventional method; these patients underwent subsequent BLT cryoablation. Thus, 25 patients underwent subsequent BLT cryoablation using contrast media, and all patients exhibited optimal occlusion. Occlusion grade 4 was observed in 56.0% (14/25) of patients, and grade 3 was observed in 44.0% (11/25) of patients during the BLT. Furthermore, the occlusion grade was improved with BLT compared to the conventional method in 92.0% (23/25) of patients.

Right inferior pulmonary vein isolation was achieved using conventional method and subsequent BLT CB ablation in 89.2% (83/93) of the patients (blue or green panels). Additional ablations were required in 10.8% (10/93) of the patients after the protocol (yellow panels). Two patients underwent additional conventional CB ablation, and eight patients underwent touch‐up ablations followed by RIPV isolation. Among the eight patients, two underwent touch‐up ablations at the bottom, four at the upper site, one at the posterior site, and one at both the bottom and posterior sites of the RIPVs.

### 3.3 Procedural and anatomical parameters in the final RIPV CB ablation

Procedural and anatomical parameters in the final RIPV CB ablation in the protocol are summarized in Table 2. Conventional CB ablation was performed in 25 patients and BLT CB ablation in 68 patients. Pressure‐guided cryoablation was performed in 60.0% (15/25) of patients undergoing conventional cryoablation and in 63.2% (43/68) of patients undergoing BLT cryoablation. The freeze duration for the final RIPV cryoablation was <180 s in 16.1% (15/93) of the patients. The balloon temperature reached −60°C within 180 s, and freezing was terminated with passive deflation in these patients. The freeze duration was 180 s in 78.5% (73/93) of the patients and 240 s in 5.4% (5/93). Rapid cooling was observed in 94.6% (88/93) of the patients. Thawing time could not be measured in two patients in the BLT group because a significant decrease in the CMAP amplitude was observed, and freezing was terminated with active deflation. We could confirm PV potentials in only 23.7% (22/93) of patients during the final RIPV cryoablation, in whom the time to isolation was measured. A time to isolation of ≤60 s was observed in 81.8% (18/22) of these patients. There were no significant differences in procedural parameters between patients who underwent conventional cryoablations and those who underwent BLT cryoablations. Anatomical parameters were investigated among 79 patients who underwent preprocedural contrast‐enhanced CT but could not be examined in the other 14 patients who underwent preprocedural plain CT. Compared to patients undergoing conventional cryoablation, those undergoing BLT cryoablation showed a larger RIPV frontal angle and sharper RIPV trans‐septal frontal angle on the coronal CT image, indicating a more inferior RIPV orientation. Meanwhile, no difference was observed in RIPV diameter, area, ovality index, right middle PV, or left atrial volume.

**TABLE 2:** Procedural and anatomical parameters in the final RIPV CB ablation in the protocol.

Variables	All	Conventional cryoablation	BLT cryoablation	*p*
	*n* = 93	*n* = 25	*n* = 68	
Freeze duration				.27
<180 s	15 (16.1)	3 (12.0)	12 (17.6)	
180 s	73 (78.5)	22 (88.0)	51 (75.0)	
240 s	5 (5.4)	0 (0.0)	5 (7.4)	
Rapid cooling	88 (94.6)	25 (100)	63 (92.6)	.20
Nadir balloon temperature (°C)	−52.5 ± 4.85	−52.7 ± 4.23	−52.4 ± 5.09	.78
Freezing time to −20°C (s)	22 (20–25)	24 (21–25)	22 (20–24)	.069
Freezing time to −40°C (s)	43 (39–50)	47 (40–50)	42 (37–50)	.29
Thawing time to 0°C (s)	10 (8–12) (*n* = 91)	10 (9–13)	9 (7–12) (*n* = 66)	.12
Thawing time to 20°C (s)	60 (51–70) (*n* = 91)	58 (52–68)	61 (51–70) (*n* = 66)	.50
Time to isolation ≤60 s	18 (81.8) (*n* = 22)	4 (80.0) (*n* = 5)	14 (82.4) (*n* = 17)	.68
RIPV max diameter (mm)	16.9 ± 2.68 (*n* = 79)	17.2 ± 3.20 (*n* = 24)	16.7 ± 2.45 (*n* = 55)	.49
RIPV minimal diameter (mm)	12.2 ± 3.20 (*n* = 79)	12.6 ± 3.91 (*n* = 24)	12.0 ± 2.86 (*n* = 55)	.46
RIPV area (mm^2^)	614.5 (475.6–790.8) (*n* = 79)	632.1 (474.0–802.6) (*n* = 24)	614.5 (496.7–757.5) (*n* = 55)	.80
RIPV ovality index	1.34 (1.18–1.61) (*n* = 79)	1.25 (1.16–1.70) (*n* = 24)	1.36 (1.21–1.59) (*n* = 55)	.49
RIPV frontal angle (°)	8.05 ± 10.9 (*n* = 79)	2.55 ± 7.73 (*n* = 24)	10.4 ± 11.2 (*n* = 55)	.002
RIPV TS frontal angle (°)	48.4 ± 11.2 (*n* = 79)	54.4 ± 9.17 (*n* = 24)	45.7 ± 11.1 (*n* = 55)	.001
RIPV transversal angle (°)	36.7 ± 14.5 (*n* = 79)	36.6 ± 13.1 (*n* = 24)	36.7 ± 15.2 (*n* = 55)	.96
RIPV TS transversal angle (°)	98.5 ± 14.4 (*n* = 79)	99.4 ± 11.5 (*n* = 24)	98.0 ± 15.6 (*n* = 55)	.69
Right middle pulmonary vein	11 (13.9) (*n* = 79)	2 (8.3) (*n* = 24)	9 (16.4) (*n* = 55)	.29
Left atrial volume (ml)	102.9 (89.1–121.8) (*n* = 79)	100.1 (86.1–121.3) (*n* = 24)	104.5 (89.7–122.8) (*n* = 55)	.89
Left atrial volume index (ml/m^2^)	62.4 (52.9–74.2) (*n* = 79)	59.7 (51.7–78.0) (*n* = 24)	63.0 (53.6–74.0) (*n* = 55)	.87

*Note*: Values are mean ± SD, number of patients (%) or median (interquartile range). RIPV ovality index is determined based on the ratio between the maximal and minimal ostial diameter.

Abbreviations: BLT, balloon lever technique; CB, cryoballoon; RIPV, right inferior pulmonary vein; SD, standard deviation; TS, trans‐septal.

## 4 DISCUSSION

This study found that BLT was effective when optimal occlusion, rapid cooling, or PV isolation could not be achieved using the conventional method during RIPV CB ablation.

### 4.1 Outcome of RIPV CB ablation and touch‐up

A previous study reported lower incidences of complete RIPV occlusion and acute success in RIPV cryoablation than in other PVs.^4^, ^12^ Furthermore, the proportion of PVs requiring touch‐up ablations was highest in RIPV. The touch‐up rate of the RIPV ranged from 16% to 20%, and the most common touch‐up ablation site was the bottom of RIPV.^3^, ^4^, ^13^, ^14^ The incidence of late reconnections was also highest in the RIPV, and the most frequent reconnection site was reported to be the bottom^3^, ^11^, ^12^, ^14^, ^15^, ^16^, ^17^, ^18^ because of the anatomical challenges which make the contact of the CB with the bottom of the RIPV difficult. In the present study, RIPV was isolated with BLT in 85.3% (58/68) of patients in whom the conventional method was ineffective. Furthermore, additional ablations after the protocol and touch‐up ablation were required in 10.8% (10/93) and 8.6% (8/93) of patients, respectively, which were lower than the RIPV touch‐up rates reported in the previous studies. Meanwhile, in the Cryo AF Global Registry in Japan, the touch‐up rate was 6.5% (23/352) of the patients,^19^ which is lower than the RIPV touch‐up rate in this study. Touch‐up ablation may have been further reduced if additional CB ablations were performed using the techniques described below. Among the eight patients requiring touch‐up ablations, four underwent touch‐up ablations at the upper site of the RIPV. Conventional CB ablations were performed prior to the BLT in three patients, which showed slow cooling. Another patient underwent BLT cryoablation without conventional ablation. The pressure to the upper site might have decreased during the BLT in these four patients because the CB was maneuvered downwards with the BLT. However, we could not confirm contact of the balloon with the upper site in all four patients because cryoablation was performed without contrast media. Meanwhile, only three patients underwent touch‐up ablations at the bottom of the RIPV. The BLT may increase the possibility of successful contact of the CB at the bottom of the RIPV, which would contribute to optimal occlusion.

### 4.2 Procedural and anatomical parameters involved in RIPV isolation

The optimal PV occlusion with the CB was reported to be the determinant of lower nadir balloon temperature, shorter freezing time to a specific temperature, shorter time to the PVI, and a longer thawing time, and these factors were associated with late PV reconnections.^12^, ^13^, ^15^, ^16^, ^18^, ^20^ Regarding the RIPV, another study reported that complete occlusion contributed to the corresponding procedural parameters and the acute success of RIPV isolation, although complete occlusion was achieved in only 33.9% of RIPVs.^4^ Furthermore, a longer thawing time and lower nadir CB temperature were reported to predict the higher durability of RIPV isolation.^13^ In the present study, 94.6% (88/93) of the patients showed rapid cooling during the final CB ablation (Table 2). The nadir balloon temperature, freezing time to −20 and −40°C, and thawing time were sufficient for achieving durable PVI. Furthermore, the nadir balloon temperature was significantly lower, the freezing time was significantly shorter, and the thawing time was significantly longer during subsequent BLT than during initial conventional cryoablation, as shown in Figures 5 and 6. These phenomena may be attributed to the optimal occlusion achieved with the BLT. Regarding anatomical parameters, a previous study reported that a more inferior‐oriented RIPV predicted late electrical RIPV reconnection after CB ablation; the reconnection site was at the bottom in 76% of patients.^11^ In the present study, an inferior RIPV orientation was observed in patients who underwent ineffective conventional methods with subsequent BLT cryoablation (Table 2). The contact of the CB with the bottom may be especially difficult in cases of an inferior‐oriented RIPV using the conventional method, and the BLT may be useful for contacting the bottom of the inferior‐oriented RIPV.

### 4.3 Technique for RIPV optimal occlusion

Chun et al. reported the “single big cryoballoon technique”, which included the “hockey stick technique”, the “pull‐down technique”, and the “big loop technique”, using first‐generation CBs.^6^ Martins et al. reported a systematic stepwise approach for RIPV CB ablation.^5^ Another study reported the “Reversed U‐curve maneuver” and the “Boutonnière‐like maneuver”. The former resembles the “hockey stick technique”, and the latter requires the insertion of the Achieve catheter into the superior branch of the RIPVs.^11^ If these techniques were used for RIPV CB ablation, the touch‐up rate might have been lower in the present study. However, RIPV CB ablation using the “single big cryoballoon technique” required three or more CB applications in 63.0% (17/27) of the patients, while only 8.6% (8/93) of the patients underwent three or more CB applications in our study. The systematic stepwise approach, the “Reversed U‐curve maneuver”, and “Boutonnière‐like maneuver” required a relatively complicated procedure, while the BLT only required the lever to be pressed forward. Thus, BLT is more straightforward and less complicated than previously reported techniques, which may be an advantage of BLT.

### 4.4 Study limitations

This study has some notable limitations. First, this was a single‐center retrospective study, resulting in the possibility of selection bias. Second, poor occlusion was observed in 45.2% (42/93) of patients, while occlusion grade 1 or 2 was observed in 57.1% (20/35) of patients using the conventional method, which was considered high. We used BLT cryoablation immediately after the ineffective conventional method. We spent limited time on the conventional method, whose proficiency may have been insufficient, especially in patients with the inferior RIPV orientation. Therefore, BLT CB ablation may have been applied to the RIPVs that were likely to be isolated using the prolonged conventional method. Third, the optimal occlusion rate during the BLT could not be investigated retrospectively in patients undergoing CB ablation without contrast media. However, optimal occlusion should have been achieved in almost all patients undergoing BLT cryoablations because the procedural parameters of the BLT were comparable with those of conventional cryoablation with optimal occlusion, as shown in Table 2. Fourth, time to isolation could be measured in only 23.7% (22/93) of patients. We positioned the Achieve catheter deep in the inferior branch and made no effort to record RIPV potentials. Fifth, late RIPV reconnection and clinical recurrence rates were not investigated. Large‐scale prospective studies are needed to address these issues.

## 5 CONCLUSIONS

Right inferior pulmonary vein isolation was achieved with BLT in approximately 85% of patients in whom the conventional method was ineffective. Thus, the BLT should be useful when RIPV is not isolated using the conventional method. This phenomenon may be attributed to the optimal occlusion with the BLT, which is caused by the effective contact of the CB with the bottom of the RIPV.

## CONFLICT OF INTEREST

The authors declare that they have no conflict of interest.

## DECLARATIONS

*Approval of the research protocol*: The study protocol was approved by the ethics committee of Matsumoto Kyoritsu Hospital. *Informed consent*: For this type of study, formal consent is not required. *Registry and the registration number*: N/A. *Animal studies*: N/A.

## Data Availability

The data from which the findings of this study were inferred can be obtained from the author upon reasonable request.
